# Free-Standing, Interwoven Tubular Graphene Mesh-Supported Binary AuPt Nanocatalysts: An Innovative and High-Performance Anode Methanol Oxidation Catalyst

**DOI:** 10.3390/nano12101689

**Published:** 2022-05-16

**Authors:** An T. Nguyen, Van Viet Tran, Asnidar Siahaan, Hung-Chih Kan, Yung-Jung Hsu, Chia-Chen Hsu

**Affiliations:** 1 Department of Physics, National Chung Cheng University, Chiayi 621, Taiwan; anhydrit210@gmail.com (A.T.N.); viettran.apc@gmail.com (V.V.T.); asnidarsiahaan2014@gmail.com (A.S.); phyhck@ccu.edu.tw (H.-C.K.); 2 Department of Materials Science and Engineering, National Yang Ming Chiao Tung University, Hsinchu 30010, Taiwan; yhsu@cc.nctu.edu.tw

**Keywords:** direct methanol fuel cells, tubular graphene, supporting material, binary AuPt nanoparticles, anode catalyst, CO poisoning effect

## Abstract

Pt-based alloy or bimetallic anode catalysts have been developed to reduce the carbon monoxide (CO) poisoning effect and the usage of Pt in direct methanol fuel cells (DMFCs), where the second metal plays a role as CO poisoning inhibitor on Pt. Furthermore, better performance in DMFCs can be achieved by improving the catalytic dispersion and using high-performance supporting materials. In this work, we introduced a free-standing, macroscopic, interwoven tubular graphene (TG) mesh as a supporting material because of its high surface area, favorable chemical inertness, and excellent conductivity. Particularly, binary AuPt nanoparticles (NPs) can be easily immobilized on both outer and inner walls of the TG mesh with a highly dispersive distribution by a simple and efficient chemical reduction method. The TG mesh, whose outer and inner walls were decorated with optimized loading of binary AuPt NPs, exhibited a remarkably catalytic performance in DMFCs. Its methanol oxidation reaction (MOR) activity was 10.09 and 2.20 times higher than those of the TG electrodes with only outer wall immobilized with pure Pt NPs and binary AuPt NPs, respectively. Furthermore, the catalyst also displayed a great stability in methanol oxidation after 200 scanning cycles, implying the excellent tolerance toward the CO poisoning effect.

## 1. Introduction

Direct methanol fuel cells (DMFCs) have received high attention among various power sources because of their high power efficiency, ultra-low pollution, low noise, high reliability, low operating temperature, and easy to maintain and handle [1,2,3,4,5]. However, DMFCs still suffer drawbacks such as carbon monoxide (CO) poisoning effect, and slow anode kinetics that may limit their industrial applications [6,7]. High loadings of platinum (Pt) and Pt-based materials offer good catalytic activity, chemical stability, and high exchange current density. Therefore, they are most commonly used as anode catalysts in DMFCs [8,9,10]. Nevertheless, the large utilization of Pt entails high intrinsic costs and poor durability of the fuel cell systems. Pt-based alloys or bimetallic catalysts, which offer better long-term stability and lessen the Pt dependency, have been developed to promote DMFC performance [11,12,13]. Transition metals such as ruthenium (Ru), palladium (Pd), and gold (Au), etc., are well matched to Pt and can play a role as CO poisoning inhibitors on Pt [14,15,16]. These second metals can significantly increase electrochemical active surface areas of Pt [17] and promote the formation of metal-OH groups, which boost the oxidation of CO species adsorbed on Pt to CO_2_, subsequently reactivating Pt active sites [18,19].

Moreover, the performance of DMFCs can also be enhanced by improving catalyst dispersion and innovating the performance of supporting materials of electrocatalysts. It is well-known that an ideal supporting material requires good electrical conductivity, high chemical inertness, large surface area, good interaction with the catalyst, easy recovery, and good water handling capability to avoid flooding [20,21]. A good catalyst-support interaction can enhance catalytic performance, decrease catalytic loss, and govern charge transfer [22]. Graphene materials have great potential as a supporting material for DMFCs because of its large surface area, good conductivity, great chemical stability, and good metal-support interaction [23,24,25,26,27]. Nevertheless, planar graphene sheets are easy to restack due to attractive force, and lead active surface area to be limited in two dimension (2D) [20,27]. On the other hand, reduced graphene oxide possesses a poor conductivity compared to chemical vapor deposition graphene [28,29], thus resulting in a degradation of electrochemical activity.

Our previous works [29,30,31] presented a free standing, flexible, and macroscopic interwoven tubular graphene (TG) mesh with superior surface area and excellent conductivity. In this work, the superior TG mesh was employed as a novel supporting material to decorate binary AuPt nanoparticles (NPs) for DMFCs. We showed binary AuPt NPs can be easily immobilized on both inner and outer walls of the TG mesh with controllable mass loading, highly dispersive and homogeneous distribution. Furthermore, we demonstrated our AuPt NPs-decorated TG mesh catalyst exhibited much superior methanol oxidation reduction (MOR) activity (*J_pf_* = 12.92 mA/cm^2^), comparing with other graphene-supported catalysts, e.g., graphene oxide-supported PtAu nanoalloy.

## 2. Experimental Section

### 2.1. Preparation of the Catalyst Supporting Material: TG Meshes

The catalyst supporting material TG meshes were prepared as followings. First, graphene-covered Ni (TG/Ni) meshes were fabricated via a thermal annealing method using cellulose acetate (CA) membrane (C045A047A, Advantec Toyo, Tokyo, Japan) as a solid carbon precursor with CA/Ni ratio of 1/4 in area; the experiment was processed at 950 °C for 8 min with heating rate of 15 °C/s under a low vacuum level of 0.4–0.8 × 10^−3^ Torr by an infrared lamp annealing system (Mila 5000, Ulvac, Advance Riko, Yokohama, Japan) as reported in our previous works [29,30,31,32,33,34]. To protect the graphene structure, the TG/Ni meshes with an area of 2 × 2 cm^2^ were immersed in a solution of poly (methyl methacrylate) (PMMA) diluted in acetone (V_PMMA_/V_acetone_ = 1:2) for 30 min, then dried in a fume hood at 50 °C for 2 h.

### 2.2. Catalysts Loading on the Supporting Material

The followings are the processes to load Au, Pt and binary AuPt NPs on the supporting material.

#### 2.2.1. Loading Au NPs on TG Meshes

Au NPs were decorated onto TG outer surfaces of TG/Ni meshes through a chemical reduction process [35]. Briefly, TG/Ni meshes with the PMMA protection layer were immersed in a reaction solution containing 5 mL of deionized water, 2.5 mL of absolute ethanol, and 100 μL of 0.1 M NaOH solution, followed by separately adding different volumes (60 μL, 90 μL, 120 μL, and 180 μL) of 10 mM HAuCl_4_ solution. Au NPs were successfully grown on outer surfaces of TG/Ni meshes as the meshes were gradually stirred at 50 °C for 3 h. Subsequently, these Au NPs-decorated TG/Ni meshes were first immersed in an etching solution of FeCl_3_/HCl (1M/1M) overnight to remove Ni wires, then they were carefully washed several times by deionized water. Finally, TG meshes loaded with different weights of Au NPs (TG/Au*_x_*) were obtained after thermal removal of PMMA at 450 °C for 30 min under a low vacuum condition. Note that *x* in TG/Au*_x_*denotes the loading mass (mg) of Au on TG.

#### 2.2.2. Loading Pt NPs on TG Meshes

Pt NPs were decorated onto TG outer surfaces of TG/Ni meshes through a chemical reduction process using NaBH_4_ as a reducing reagent. The TG/Ni meshes with the PMMA protection layer were immersed into a solution containing 5 mL of deionized water, 2.5 mL of absolute ethanol, and 100 μL of 0.1 M NaOH. The mixture solution was kept at 50 °C, followed by separately adding different volumes (60 μL, 75 μL, 90 μL, and 120 μL) of 10 mM H_2_PtCl_6_ solution. Subsequently, 1 mL of 1.0 M NaBH_4_ dissolved in deionized water was gradually dropped into the solutions for the reduction of Pt molecular precursors to grow Pt NPs. After being stirred for 2 h, Pt NPs were successfully formed on the outer surfaces of all TG/Ni meshes. TG meshes loaded with different weights of Pt NPs (TG/Pt*_y_*) were obtained after the Ni etching and the PMMA removal steps as mentioned in the TG/Au_*x*_ fabrication process. Note that *y* in TG/Pt*_y_* represents the loading mass (mg) of Pt on TG.

#### 2.2.3. Loading Binary AuPt NPs on TG Meshes

To obtain better distribution of binary AuPt NPs on TG meshes, we first decorated Au NPs on outer surfaces of TG/Ni meshes following the procedures used to load Au NPs on TG/Ni meshes, except that only 90 μL of HAuCl_4_ solution was used here. The reason of choosing 90 μL of 10 mM HAuCl_4_ solution will be explained in the results and discussion section. After the decoration of Au NPs on outer surfaces of TG/Ni meshes, different volumes (60 μL, 75 μL, 90 μL, and 120 μL) of 10 mM H_2_PtCl_6_ solution were separately added into the mixed solution. Then the procedures used to load Pt NPs onto TG/Ni meshes were used to grow binary AuPt NPs onto TG/Ni meshes. Finally, the Ni etching and the PMMA removal steps were processed to obtain TG meshes loaded with binary AuPt NPs onto outer surfaces of TG meshes, denoted as TG/Au*_x_*Pt*_y_*, where *x* and *y* are the loading mass percentages of Au and Pt NPs on TG, respectively.

In addition, we also decorated binary AuPt NPs on both outer and inner surfaces of TG meshes. To achieve that, hollow cylindrical structure of graphene-covered Ni meshes (see Figure S1a) were prepared. First, TG/Ni meshes, after cutting their edges, were immersed in a solution of FeCl_3_/HCl (1M/1M) for 10 min to etch part of Ni wires. The hollow cylindrical structure of one of the TG/Ni meshes can be clearly observed through the electronic transparency of the SEM image as displayed in Figure S1b. The average diameter of Ni cores was approximately 37 ± 5 μm, whereas the original diameter was approximately 50 μm. Binary AuPt NPs were loaded on both surfaces of the TG meshes following the aforementioned procedures. The prepared sample is denoted as TG’/Au*_x_*Pt*_y_*, where *x* and *y* are loading mass percentages of Au and Pt on TG’. TG’ represents both inner and outer walls of the TG mesh which are immobilized with Au and Pt NPs. To exclude the Fe traces, after the etching process, the samples were immersed in DI water for 6 h and repeated it for three times.

### 2.3. Characterization

The morphology and distribution of noble metal NPs decorated on TG meshes were characterized with a field-emission scanning electron microscope (FESEM, Hitachi S4800-I, Tokyo, Japan) and an ultrahigh resolution analytical electron microscope (HRAEM, JEOL-2100F, Tokyo, Japan, operated at 200 kV). BET area of TG mesh was measured by a specific surface area and pore size distribution analyzer (Micromeritics, ASAP2020, Norcross, GA, USA). A Raman spectroscope (Horiba, XploRA ONE Tokyo, Japan) and an X-ray diffractometer (XRD, Bruker Smart APEX CCD, Madison, WI, USA) were used to investigate the crystallographic structures of the samples. The elemental analysis was conducted with energy-dispersive X-ray spectroscopy (EDS) of the Hitachi S4800-I, JEOL-2100F and JEOL JEM-2010. The chemical states of the Au (Pt) decorated TG meshes were examined with an X-ray photoelectron spectroscope (XPS, Kratos Axis Ultra DLD, Manchester, UK). The masses of metals loaded on TG were measured by inductively coupled plasma-mass spectrometer (ICP-MS, THERMO-ELEMENT XR, Waltham, MA, USA). A four-point probe instrument (LRS4-TG, KeithLink Technology, Taipei, Taiwan, and Keithley 2636B, Solon, OH, USA) was used to measure the sheet resistance of the samples at room temperature. Electrochemical measurements were conducted with electrochemical workstations (Jiehan 5600, Jiehan Technology, Taichung, Taiwan and Autolab PGSTAT204, Metrom Autolab B.V., Utrecht, The Netherlands) using a three-electrode system containing a working electrode (e.g., TG/AuPt catalyst), Pt counter electrode, and an Ag/AgCl/KCl reference electrode. Note that the areas of all work electrodes were fixed at 2 × 2 cm^2^. The CV profiles of methanol oxidation were measured in a solution of 0.5 M KOH + 1 M CH_3_OH on the TG-based catalysts with the scan rate of 20 mV/s. Electrochemical impedance spectroscopy (EIS) measurements were performed on all TG (TG’)/AuPt catalysts in a solution of 0.5 M KOH + 1 M CH_3_OH at an applied voltage of 0.3 V with a frequency range from 0.1 to 10^5^ Hz by an electrochemical workstation (Autolab PGSTAT204).

## 3. Results and Discussion

### 3.1. Material Characterization

**Figure 1:**
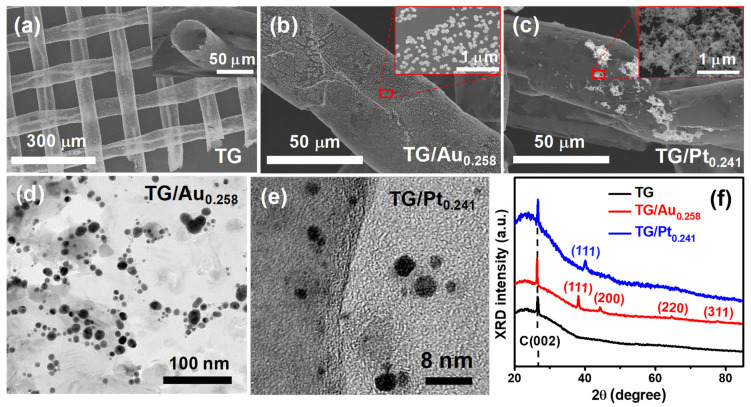
(**a**) SEM image of a TG mesh and a magnified graphene tube in the inset; (**b**,**c**) SEM images of the TG/Au_0.258_ and TG/Pt_0.241_, respectively; (**d**,**e**) Corresponding TEM images of (**b**,**c**), respectively. (**f**) XRD patterns of the TG, TG/Au_0.258_, and TG/Pt_0.241_.

Figure 1a shows the SEM image of an interwoven TG mesh with diameters of approximately 50 µm and thin wall edges. The insert displays a magnified morphology of an open-ended graphene tube. Carbon impurities resulted from the thermal annealing process appeared as a small wire in the core of each tube [30]. The freestanding TG meshes were chosen as a supporting material for the decoration of noble metal NPs catalysts because of their low sheet resistance (48 Ω/sq) [29] and high surface-to-volume ratio provided by their three-dimensional structure. The BET area of the interwoven TG mesh is about 364.4 m^2^·g^−1^ measured by a specific surface area and pore size distribution analyzer. The former can offer high electrical conductivity (~1.48 × 10^6^ S·m^−1^) [30] while the latter can provide more active surface area to grow catalysts. Hence, the freestanding TG meshes should be exceptionally suitable to serve as a supporting material for electrocatalytic applications.

Table S1 shows the mass loadings of Au NPs on a fixed area (2 × 2 cm^2^) of the TG/Au*_x_* catalysts determined by ICP-MS analysis. Figure S2 displays the FE-SEM images of TG/Au meshes obtained with different volumes of HAuCl_4_ solutions. It is clear to see that Au NPs were uniformly distributed on the TG surfaces for both TG/Au_0.171_ and TG/Au_0.258_, while for the cases of TG/Au_0.346_ and TG/Au_0.554_, Au NPs were aggregated, especially for the TG/Au_0.554_, due to the abundance of Au NPs. Furthermore, compared to the TG/Au_0.171_, the TG/Au_0.258_ possessed a higher surface coverage of Au NPs. Therefore, the TG/Au_0.258_ was chosen for further investigations. Figure 1b and its inset show SEM images of the TG/Au_0.258_ catalyst which clearly display the uniform decoration of Au NPs on the outer wall of the TG. The morphology and distribution of Au NPs were further examined in the TEM image shown in Figure 1d. Sizes of Au NPs were in the range from 3.3 to 14.4 nm, determined by counting over 30 particles. On the other hand, Figure 1c shows that Pt NPs were not uniformly distributed on the TG surface of the TG/Pt_0.241_ catalyst, evidenced by strong aggregation of a numerous amount of Pt NPs to form nondispersive Pt clusters (see Figure 1c). This is due to the ultra-flat surface and scarce functional groups of the TG [36]. Pt NPs had diameters of 2 to 4 nm, which were much smaller than those of Au NPs (see Figure 1e).

Figures S3 and S4 show EDS analyses of the TG/Au_0.258_ and TG/Pt_0.241_ catalysts, respectively, which reveal the composition of noble metal NPs and confirm the complete removal of Ni after the etching process. Au NPs were uniformly decorated on the surface of TG/Au_0.258_ catalyst with the concentration of 6.37 wt.% (see Figure S3). Moreover, for the TG/Pt_0.241_ catalyst, EDS analyses were acquired at two different positions because of the inhomogeneous distribution of Pt clusters. At high coverage area, Pt concentration was 21.0 wt.% (Figure S4a,b), whereas at low coverage area, Pt concentration was 7.7 wt.% (Figure S4c,d). Significant amounts of oxygen existed in both samples were originated from the Ni wet etching process and it is confirmed by the EDS analysis, obtained from the TG/Pt_0.241_ catalyst before Ni etching, where no oxygen was found (Figure S5). XRD measurement was performed to identify crystallographic structures of the TG mesh, TG/Au_0.258_, and TG/Pt_0.241_ catalysts (see Figure 1f). The sharp peaks at approximately 26.4° in the three XRD patterns are attributed to the hexagonal phase of graphite (see the PDF#41-1487), which is originated from the multi-layered feature of the TG mesh [36]. The main peaks (111) in the XRD patterns of the TG/Au_0.258_ and TG/Pt_0.241_ are approximately at 38.23 and 39.90°, respectively. Moreover, XRD patterns of the TG/Au_0.258_ and TG/Pt_0.241_ reveal that both Au and Pt NPs are face-centered cubic metal compounds according to the reference PDF#04-0784, and PDF#04-0802, respectively.

**Figure 2:**
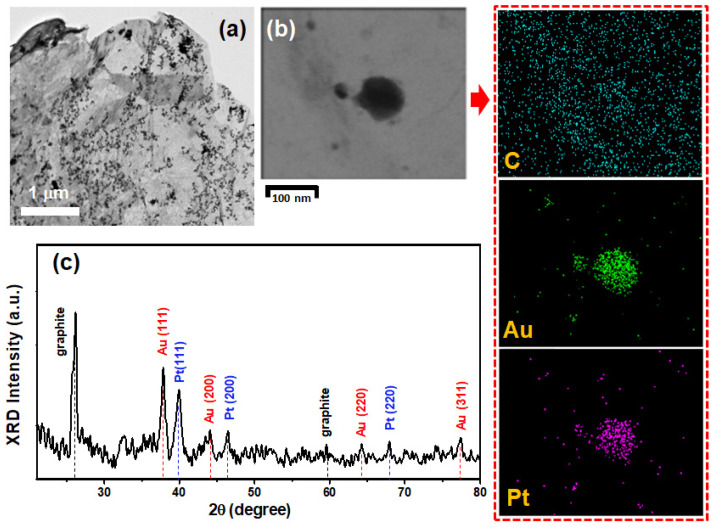
Characterization of the TG/Au_52_Pt_48_ catalyst: (**a**) TEM image; (**b**) TEM-EDS mapping images for C, Au, and Pt; and (**c**) XRD pattern.

Figure 2a shows the TEM image of the TG/Au_52_Pt_48_ catalyst. The image reveals that binary AuPt NPs are dispersive and homogenously distributed on the surface of TG, in contrast to the nondispersive distribution of Pt clusters of the TG/Pt_0.241_ catalyst. Table 1 shows the mass loadings, mass loading percentages, and atomic ratio of Au and Pt NPs on a fixed area (2 × 2 cm^2^) of the TG/Au*_x_*Pt*_y_* catalysts determined by ICP-MS analysis. As indicated, the mass loadings of Au and Pt NPs on TG meshes can be controlled by changing the adding volumes of HAuCl_4_ and H_2_PtCl_6_ precursor solutions, respectively. The mass loading of metal (Au, Pt) on TG increased with the adding volume of precursor solution. Table 1 also displays average sizes of binary AuPt NPs of different catalysts determined by counting over 30 particles from TEM images. As indicated, average sizes of binary AuPt NPs increased with the adding volume of Pt precursor solution because more Pt atoms were supplied for the formation of binary NPs. The EDS analysis of the TG/Au_52_Pt_48_ catalyst is displayed in Figure S6, obtained by scanning on a large area of 3.7 × 4.9 μm^2^. It indicates that the element distribution of Au (12.74 wt.%) and Pt (11.33 wt.%) were very close and it is consistent with the ICP-MS result. Moreover, no Fe element was found in the EDS analyses (Figures S3, S4 and S6), confirming the total removal of Fe traces. The TEM-EDS element-mapping data of the TG/Au_52_Pt_48_ catalyst (Figure 2b) shows a spatial well overlapping of Au and Pt elements in both big particle (~100 nm) and small particle (~20 nm), indicating that Pt NPs grew at Au NP sites to form binary AuPt mixing NPs [17,36]. The TEM-EDS element-mapping result also rules out the formation of the core-shell Au@Pt NPs. Furthermore, the TEM-EDS analyses (see Figure S7) obtained from three different NPs provided another evidence for the formation of binary AuPt mixing NPs within the solid matrix. During the binary AuPt mixing NPs formation process, Au NPs served as growth centers and stabilizers of Pt NPs [36] and thus Pt NPs were automatically grown at Au NP sites [17]. The formation of binary AuPt mixing NPs on TG meshes is paramount for the further improvement of the electrocatalytic performance of DMFCs which will be discussed later.

XRD pattern of the TG/Au_52_Pt_48_ catalyst (Figure 2c) shows sharp diffraction peaks at approximately 26.4° and 59.7°, which are assigned to hexagonal phase of graphite (see the PDF#41-1487). Furthermore, it also exhibits the characteristic peaks associated to the (111), (200), (220), and (311) lattice planes of face-centered cubic Au (PDF#04-0784) and the (111), (200) and (220) lattice planes of face-centered cubic Pt (PDF#04-0802), indicating the formation of Au and Pt crystal structures simultaneously [37,38]. The sharp peaks (200) and (220) of Pt, which were not observed in the TG/Pt_0.241_ catalyst, can clearly be observed in the TG/Au_52_Pt_48_ catalyst. This represents that higher crystallinity of Pt was formed in the TG/Au_52_Pt_48_ catalyst. The main peaks (111) in the XRD patterns of the TG/Au_52_Pt_48_ were not merged (separated at 38.23 and 39.90°, respectively), suggesting no formation of Pt and Au alloy [39]; it should be just a mixture formation of Pt and Au.

**Figure 3:**
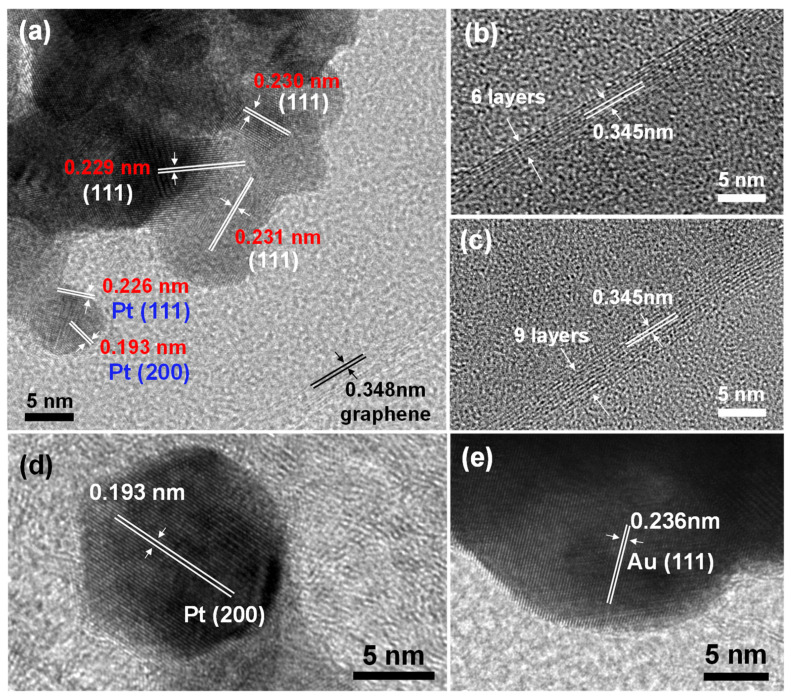
(**a**) HRTEM images of the TG/Au_52_Pt_48_; (**b**,**c**) HRTEM images taken on the wall-edges of the TG/Au_52_Pt_48_ at different areas; (**d**,**e**) HRTEM images of the TG/Pt_0.241_ and TG/Au_0.258_, respectively.

Figure 3 further displays the detailed crystallographic structures of the TG mesh decorated by the metallic NPs. The HRTEM image displayed in Figure 3a, taken from the TG/Au_52_Pt_48_ catalyst, clearly exhibits four distinct sets of lattice fringes. The observed interlayer distances of 0.230, 0.229, and 0.231 nm are closely in agreement with the (111) lattice spacing values of the cubic Au and Pt reported in [40], suggesting the formation of binary AuPt nanostructures. Moreover, the observed interlayer distances of 0.226 and 0.193 nm are consistent with the d-spacing of Pt (111) and Pt (200) lattice planes, respectively, as reported in [24,37]. The result indicates the presence of pure Pt NPs besides binary AuPt NPs. HRTEM images taken on the wall-edge areas (see Figure 3a–c) of the TG/Au_52_Pt_48_ catalyst directly reflected the graphene layered structure, which contains 6–9 graphene layers. Figure 3d,e show the HRTEM results acquired from the TG/Pt_0.241_ and TG/Au_0.258_ catalysts, respectively. Interlayer spacing values of 0.193, and 0.236 nm were observed, consistent with the lattice spacing values of Pt (200) and Au (111) [24,38], respectively.

**Figure 4:**
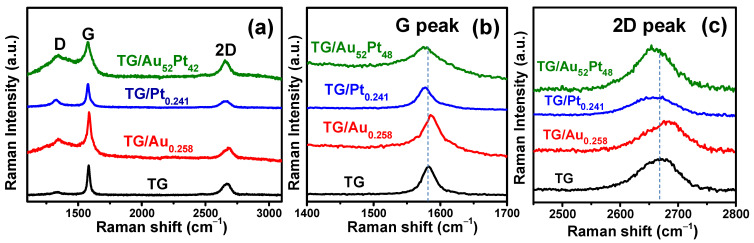
**(a)** Raman spectra of the TG, TG/Au_0.258_, TG/Pt_0.241_, and TG/Au_52_Pt_48_ catalysts; (**b**) the shift of G peak; (**c**) the shift of 2D peak.

Figure 4 shows the Raman spectra acquired on surfaces of the TG, TG/Au_0.258_, TG/Pt_0.241_, and TG/Au_52_Pt_48_ catalysts. The bare TG contained three Raman characteristic peaks: the exceedingly weak D peak at approximately 1335 cm^−1^, which implies negligible defects or impurities on the graphene surface and high quality of graphene, the sharp G peak (~1582 cm^−1^), which belongs to the E_2g_ vibrational mode of sp^2^ C–C stretching, and the 2D peak (~2672 cm^−1^), which is a second-order Raman process originated from the scattering of phonons at the zone boundary [31,41]. The intensity ratio of the 2D to G peaks, I_2D_/I_G_, was 0.41, representing multi-layer graphene [42]. This result is close to the value of our previous result [29].

Compared to the G and 2D peaks of the TG sample, those of the TG/Au_0.258_, TG/Pt_0.241_, and TG/Au_52_Pt_48_ catalysts were clearly shifted (see Figure 4b,c). The shift of Raman peak in graphene is mainly due to the introduction of mechanical strain and/or the carrier density modulation induced by charge transfer caused by decorated metal NPs [43,44]. The relative strength of the 2D and G peaks’ shift provides information of whether charge-transfer or strain effect dominates [44]. If the shift of the 2D peak is larger than that of the G peak, the mechanical strain effect is stronger than the carrier density modulation. Otherwise, the carrier density modulation effect is dominant. Moreover, the direction of the 2D peak shift gives information about doping [44,45,46,47]. If the 2D peak is red-shifted, it represents that graphene is doped by electron. On the contrary, if the 2D peak is blue-shifted, it indicates that graphene is doped by holes [44,45,46,47]. From Figure 4b,c, the 2D peak shifts of the TG/Au_0.258_, TG/Pt_0.241_, and TG/Au_52_Pt_48_ were found to be +14 cm^−1^, −11 cm^−1^, and −12 cm^−1^, respectively. On the other hand, the G peak shifts of these three catalysts were found to be +4 cm^−1^ (TG/Au_0.258_), −5 cm^−1^ (TG/Pt_0.241_), and −4 cm^−1^ (TG/Au_52_Pt_48_). It indicates the strain effect is stronger than carrier density modulation effect in these three samples, probably due to large lattice mismatch between decorated metal NPs and TG mesh [44,48]. From the blue-shifted 2D peak of the TG/Au_0.258_, occurrence of hole doping in the TG/Au_0.258_ is revealed. On the contrary, both TG/Pt_0.241_ and TG/Au_52_Pt_48_ catalysts had electron doping effect [44,47,49], since both of them had red-shifted 2D peaks. From Figure 4a, the intensity ratios between D and G peaks (I_D_/I_G_) of the TG, TG/Au_0.258_, TG/Pt_0.241_, and TG/Au_52_Pt_48_ were found to be 0.097, 0.413, 0.318, and 0.619, respectively. The increase in I_D_/I_G_ values of TG/Au_0.258_, TG/Pt_0.241_, and TG/Au_52_Pt_48_ is presumably attributed to the carrier density modulation of graphene and not due to the increase in defects [50].

**Figure 5:**
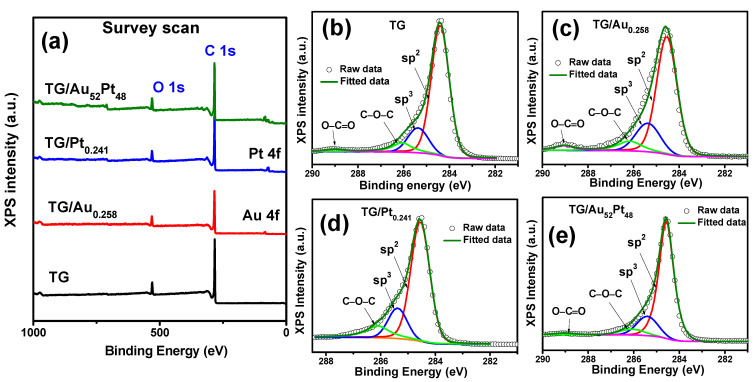
(**a**) XPS survey scans of four different TG-based catalysts, exhibiting the dominant C 1s and weak O 1S peaks. (**b**–**e**) C 1s narrow scans of the TG, TG/Au_0.258_, TG/Pt_0.241_, and TG/Au_52_Pt_48_, respectively, containing sp^2^, sp^3^ bonding, and small quantity of oxygen-attached carbon functional group.

XPS results of the samples were employed to analyze the surface chemical properties and to prove that the increase in D peak intensities after the decoration of noble metal NPs was not due to defects. The XPS survey scans of all the TG-based catalysts (Figure 5a) exhibited dominant narrow C 1s and low O 1s peaks. Figure S8 displays O 1s scans of the TG-based catalysts and it shows that oxygen concentration of all four catalysts were almost the same. It indicates that the noble metal NPs decoration process did not change oxygen concentration. Furthermore, no Ni peak was observed for all four catalysts confirming the complete removal of Ni, and it agrees with the EDS analyses. Figure 5b–e display C 1s scans of the TG, TG/Au_0.258_, TG/Pt_0.241_, and TG/Au_52_Pt_48_, respectively, which were deconvoluted into four components. The two main components appearing at binding energies of 284.4 eV and 285.4 eV are associated to carbon sp^2^ and sp^3^, respectively. TG exhibited a high sp^2^/sp^3^ ratio of approximately 4.9 implying the good quality of graphene [32,34]. The sp^2^/sp^3^ ratios of TG/Au_0.258_, TG/Pt_0.241_, and TG/Au_52_Pt_48_ were found to be 3.8, 4.2, and 4.7, respectively, and were quite close to that of the TG. This indicates that the noble metal NPs decoration process only slightly increased the defects of TG [51]. In addition, the weak components at 286.1 eV and 289.0 eV were assigned to O–C–O and O–C=O functional groups [32], respectively, representing that only a slight quantity of oxygen existed inside graphene lattices; upon the growth of noble metal NPs.

**Figure 6:**
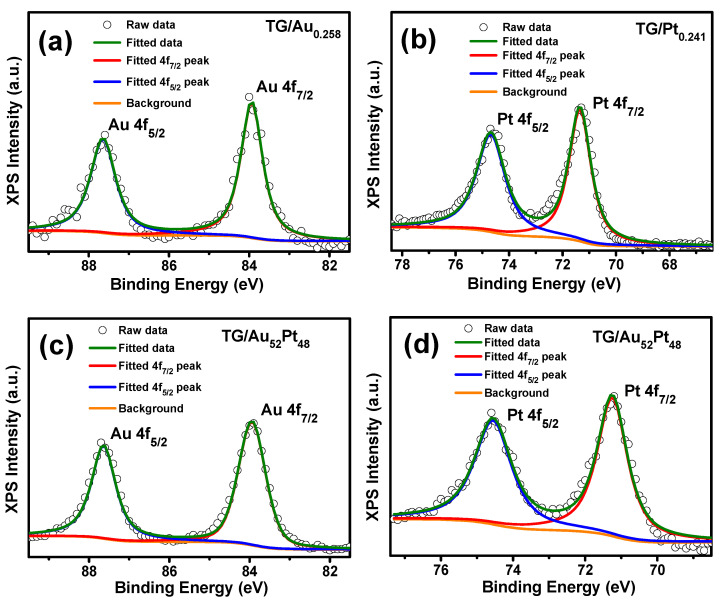
Au 4f (**a**,**c**) and Pt 4f (**b**,**d**) narrow scans of the TG decorated with noble metal NPs and their corresponding fitting components.

Au 4f and Pt 4f scans (Figure 6) of the TGs decorated with noble metal NPs were used to affirm the state of the Pt and Au, and to determine the electron transfer in binary AuPt NPs. The fitted results, shown in Figure 6, contained the intense doublet of Au (~83.9 and 87.6 eV) for the TG/Au_0.258_ (see Figure 6a), and Pt (~71.4 and 74.7) for the TG/Pt_0.241_ (see Figure 6b) owing to metallic Au^0^ and Pt^0^, respectively [15,24,36]. The Au 4f binding energies of the TG/Au_0.241_ were a little bit lower than those of the standard metallic Au^0^ [52,53] (84.0 eV for Au 4f_7/2_ and 87.7 eV for Au 4f_5/2_), due to electron transfer from graphene to Au (p-doped of graphene), which agrees with the aforementioned interpretation for the blue-shift of the 2D Raman peak of the TG/Au_0.258_ catalyst. On the other hand, the intense doublet of Pt for the TG/Pt_0.241_ (71.4, and 74.7 eV) exhibited a positive shift in binding energies compared to standard Pt^0^ metal [54,55] (Pt 4f_7/2_, 71.0 eV and Pt 4f_5/2_, 74.4 eV), which is attributed to electron transfer from Pt to graphene (n-doped of graphene). It agrees with what we proposed for the red-shift of the 2D Raman peak observed in the TG/Pt_0.241_ catalyst. As indicated in Figure 6c, the Au 4f_7/2_ and 4f_5/2_ binding energies of TG/Au_52_Pt_48_ are 83.9 eV and 87.6 eV, respectively, same as those of TG/Au_0.258_, and lower than those of standard metallic Au^0^, indicating no change of the electron structure of Au NPs after Pt NPs growth. Furthermore, lower binding energies of the Pt 4f_7/2_ (71.2 eV) and Pt 4f_5/2_ (74.6 eV) (see Figure 6d) were observed for the TG/Au_52_Pt_48_ compared with those of the TG/Pt_0.241_ (see Figure 6b), suggesting that Au NPs promote the donation of electrons from Pt to TG [17,38,56].

### 3.2. Methanol Oxidation

The Pt-based catalysts exhibit better methanol oxidation activity in alkaline environments than in acid solutions because of much weaker bonding of intermediates to the electrocatalyst in alkaline media [15]. Consequently, CV profiles of methanol oxidation were measured in a solution of 0.5 M KOH + 1 M CH_3_OH on the TG-based catalysts with the scan rate of 20 mV/s. Bimetallic Pt-based catalysts exhibit a preeminent catalytic performance compared to pure Pt-based catalysts, due to the combined synergistic strain and ligand/electronic effect [1], which can promote the oxidation of CO to CO_2_ thus reactivating Pt active sites. The overall reaction on the binary AuPt catalyst for MOR in alkaline media obeys the “bifunctional mechanism” and can be described by the following equations [57]:

Pt + CH_3_OH → Pt-CH_3_OH_ads_ (1)

Pt-CH_3_OH_ads_ + 4OH^−^ → Pt-CO_ads_ + 4H_2_O + 4e^−^ (2)

Au + OH^−^ → Au-OH_ads_ + e^−^ (3)

Pt-CO_ads_ + Au-OH_ads_ + OH^−^ → Pt + Au + H_2_O + CO_2_ + e^−^ (4)

Obviously, the methanol oxidation process using AuPt nanocatalysts produces current and CO_2_.

**Figure 7:**
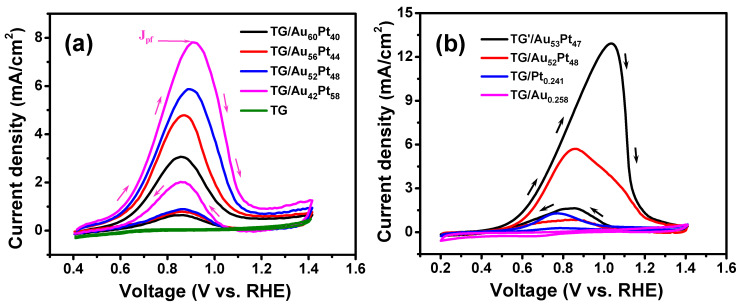
CVs of methanol oxidation on different TG-based catalysts (TG, and TG/Au*_x_*Pt*_y_*(**a**), TG’/Au_53_Pt_47_, TG/Au_52_Pt_48_, TG/Pt_0.241_ and Pt/Au_0.258_ (**b**)) measured in 0.5 M KOH + 1 M CH_3_OH solution.

Figure 7 displays CVs of methanol oxidation of different TG-based catalysts, where forward anodic peak current densities (*J_pf_*) of each CV represent the methanol oxidation activity [36,58]. As indicated in Figure 7, the TG electrode and the TG/Au_0.258_ catalyst did not exhibit MOR because of the lack of catalyst, i.e., Pt NPs. On the contrary, all CVs of all TG (TG’)/Au*_x_*Pt*_y_* catalysts exhibited well-defined forward and backward peaks. The forward peak is attributed to the oxidation of methanol molecules [14,54]. Moreover, the forward peak current density of each TG/Au*_x_*Pt*_y_* catalyst increased with the increase of Pt mass loading (see Figure 7a and Table 2). The *J_pf_* of the TG/Au_42_Pt_58_ was 7.81 mA/cm^2^, which was the highest among all the TG/Au*_x_*Pt*_y_* catalysts. This is because higher Pt mass loading led to higher surface density of Pt NPs immobilized on TGs (see Figure S9), which resulted in higher MOR activity. To enhance the MOR performance, AuPt NPs were decorated on both inner and outer surfaces of TG, leading to the highest mass loading of metals on TG. Figure S10 displays SEM images of the TG’/Au_53_Pt_47_ catalyst, which shows that binary AuPt NPs were successfully immobilized on both inner and outer surfaces of graphene tubes. The loading masses of Au and Pt on both walls of TG are approximately twice as those on outer wall (see Table 1). Figure 7b plots CVs of methanol oxidation on different TG-based catalysts. The CV curve of the TG’/Au_53_Pt_47_ catalyst exhibited the highest MOR activity (*J_pf_* = 12.92 mA/cm^2^) (see Table 2). The *J_pf_* of the TG’/Au_53_Pt_47_ catalyst were 2.20 times of that of the TG/Au_52_Pt_48_. It indicates that the decoration of binary AuPt NPs on both outer and inner surfaces of graphene tubes can enhance the MOR activity. Furthermore, the J_pf_ of the TG/Au_52_Pt_48_ catalyst were 4.59 times that of the TG/Pt_0.241_. It reveals that the immobilization of bimetallic (AuPt) NPs on TGs indeed improved the MOR activity.

**Figure 8:**
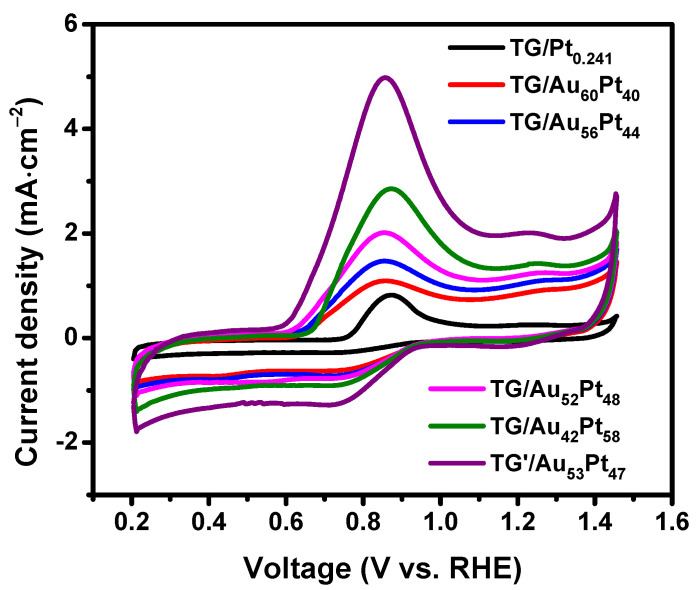
CO-stripping curves on TG-based catalysts in 0.5 M KOH.

Besides the MOR activity, the tolerance of CO or CO-like is important for electrocatalysis as well, and the onset potential of CO striping experiment is conceived to characterize the tolerance on the electrocatalytic surface [59,60,61,62]. Lower onset potential represents higher stability (i.e., reactivation efficiency) of electrocatalyst toward CO or CO-like poisoning [59,60,61,62]. To study the CO tolerance of TG/AuPt catalysts, CO stripping experiments were conducted in 0.5 M KOH solution at the scan rate of 50 mV/s (Figure 8). For CO striping experiment, CO was adsorbed on the surface of the catalysts by purging CO gas in the solution at the potential of 0.1 V (RHE) for 20 min, followed by purgation of argon gas for 30 min to remove the residue of CO. The CO stripping CV curves (Figure 8) contained the anodic peaks, resulting from the oxidation of the adsorbed CO on the surface of the catalysts [63]. Compared to TG/Pt_0.241_, the CO oxidation onset potentials (*E_onset_*) of the TG/Au*_x_*Pt*_y_* catalysts were much lower (see Table 2), indicating the importance of Au NPs on TG-based catalysts, which not only improve the growth of Pt NPs on TG but also promote the oxidization (or removal) of CO on the surface of catalyst, thus increasing the tolerance toward CO [64,65]. Moreover, *E_onset_* of TG/Au_42_Pt_58_ is the highest, compared to those of the TG/Au*_x_*Pt*_y_* catalysts. It suggests that the TG/Au_42_Pt_58_ catalyst had a low stability of electrocatalysis toward CO or CO-like poisoning. It had too many Pt NPs so that some of them were aggregated to form Pt cluster (see Figure S9d), leading to high blockage of the surface of Pt catalyst and inducing CO poisoning. This is similar to the result reported in [40]. In addition, TG/Au_52_Pt_48_ catalyst possessed the lowest value of *E_onset_* compared to those of the TG/Au*_x_*Pt*_y_* catalysts because of the well-distribution of bimetallic AuPt NPs onto tubular graphene (see Figure S9c), which could improve the particle aggregation, thus, CO poisoning effect. Moreover, the good methanol oxidation performance of the TG/Au_52_Pt_48_ catalyst is also due to the well-distribution of Pt into Au matrix, evidenced by TEM-EDS mapping in Figure 2b and well-matching of Pt and Au NPs weight percentage (see Figure S6). Au not only significantly increased the electrochemical active surface area of Pt, but also formed Au-OH groups, which boosted the oxidation of CO species adsorbed on Pt to CO_2_, subsequently reactivating Pt active sites [17,36,66]. Furthermore, comparing with TG/Au*_x_*Pt*_y_* catalysts, the TG’/Au_53_Pt_47_ catalyst had lower *E_onset_* due to its higher Au NPs mass loading and immobilization of AuPt binary NPs on both inner and outer walls of TG. The TG’/Au_53_Pt_47_ catalyst had the best CO tolerance and highest MOR performance. Based on the areas of these CO-stripping peaks, the electrochemical active surface areas (ECSAs) were evaluated by the equation: ECSA_CO_ = *Q_CO_*/(*C* × *m*), in which *Q_CO_* is the amount of charge of CO-stripping peak; *m* is the mass of Pt loaded on TG and *C* is the capacitance (for Pt, *C* value is 420 μC·cm^−2^) [67]. The calculated ECSAs are listed in Table 2. The TG’/Au_53_Pt_47_ catalyst exhibited the highest ECSA, indicating more active sites, which is consistent with CVs of MOR. A comparison of our present catalyst (TG’/Au_53_Pt_47_) with other graphene-based catalysts is summarized in Table 3. Its MOR activity is much superior to that of the other graphene-based catalysts which is attributed to the excellent conductivity and large surface area of TG mesh-supported material. Particularly, both outer and inner walls of TG can be decorated with binary Au-Pt NPs, leading to higher loadings of catalysts on TG.

**Figure 9:**
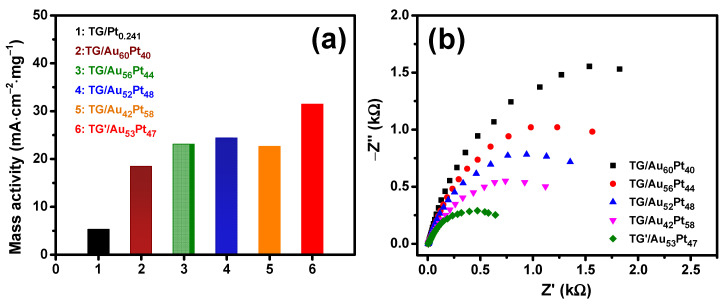
(**a**) The normalized mass activities of TG-based catalysts; (**b**) Nyquist plots of the catalysts of TG (TG’)/Au*_x_*Pt*_y_* catalysts.

To compare the intrinsic electrochemical property of TG-based catalysts, mass activities normalized by the mass of Pt are shown in Figure 9a. It clearly demonstrates that the catalytic activity of bimetallic catalysts for MOR was significantly enhanced in the mass activities compared with that of the monometallic catalyst. Moreover, the catalysts with equivalent loading mass ratio (TG/Au_52_Pt_48_ and TG’/Au_53_Pt_47_) had higher mass activity values than those of other catalysts (non-equivalent loading mass ratio), which is consistent with the trend of the onset potential values. EIS measurements were also performed on TG/AuPt and TG’/AuPt catalysts and their Nyquist plots are shown in Figure 9b, in which a smaller semicircle represents a smaller charge-transfer resistance across the electrode-electrolyte interface. It is clear that TG’/Au_53_Pt_47_ possessed the lowest charge-transfer resistance. Thus, it exhibited the highest performance toward MOR. Moreover, for other TG/AuPt catalysts, the charge-transfer resistance increased with the decrease of Pt mass loading, indicating higher Pt mass loading yielded faster reaction kinetics for MOR. Our EIS results are similar to the works reported in [64,72].

**Figure 10:**
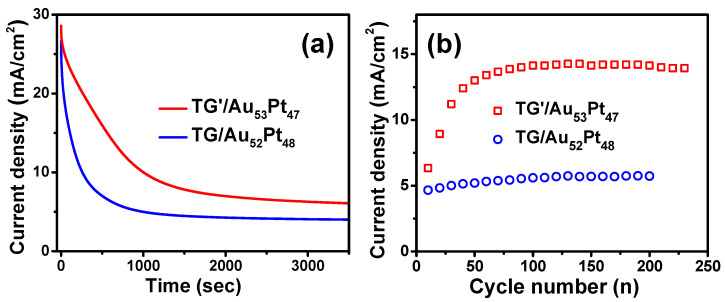
(**a**) Chronoamperometric curves measured at 1 V (vs. RHE) and (**b**) peak current densities of methanol oxidation in the forward scan vs. the cycle number of the TG/Au_52_Pt_48_ and TG’/Au_53_Pt_47_ catalysts.

At a constant potential, methanol was oxidized and the oxidized carbonaceous intermediates were adsorbed on the electrocatalytic surface which produced the poison and deactivation of the electrocatalytic surface, leading to strong initial decay of current density in the chronoamperometric curves [65]. To further investigate long-term stabilities of the TG-based catalysts, chronoamperometric curves of both TG/Au_52_Pt_48_ and TG’/Au_53_Pt_47_ catalysts were measured at the potential of 1 V (vs. RHE) in a solution of 0.5 M KOH + 1.0 M CH_3_OH. Figure 10a plots the time evolution of current densities of both catalysts. The quick decay of current densities of both catalysts at the early stage during methanol oxidation process is primarily attributed to CO poisoning effect [14,54]. Compared to TG/Au_52_Pt_48_ catalyst, the TG’/Au_53_Pt_47_ catalyst exhibited a slower decay rate due to its better CO tolerance capability. Furthermore, the TG’/Au_53_Pt_47_ catalyst also yielded a higher initial and final current densities owing to its smaller charge–transfer resistance compared to those of the TG/Au_52_Pt_48_. This suggests a better stability and superior poisoning-tolerance ability of TG’/Au_53_Pt_47_. Moreover, peak current densities of forward scans of both catalysts vs. cycle number were measured to assess their long-term stabilities in methanol oxidation (see Figure 10b). Both catalysts exhibited great stability and high efficiency in methanol oxidation after 200 scanning cycles. Figure S11 displays the CVs of methanol oxidation at cycle number 50 and 200 of both catalysts. The shapes and peak current densities of both CVs remained unchanged, indicating the high stability of both catalysts.

## 4. Conclusions

In summary, flexible and freestanding TGs were used and shown as an excellent supporting material for the decoration of noble metals. We successfully decorated binary AuPt NPs on both outer and inner walls of graphene mesh tubes, which served as catalytic anode for the enhancement of methanol oxidation in DMFCs. Compared to the TG mesh electrode with only outer wall immobilized with pure Pt NPs (or binary AuPt NPs), the MOR activity of the TG mesh electrode, whose both walls were decorated with binary AuPt NPs, was enhanced by 10.09 (or 2.20) times. In addition, its MOR activity is much superior to that of the other graphene-based catalysts which is attributed to the excellent conductivity and large surface area of TG mesh-supported material. Moreover, the catalysts with equivalent loading mass (Au/Pt) ratio had better tolerant ability toward the poisoning effect of intermediate carbonaceous species formed during the methanol oxidation than those of other catalysts (non-equivalent loading mass ratio). The results confirm that interwoven TG mesh electrode decorated with binary AuPt NPs is a great candidate as an electrocatalyst in DMFCs.

## Figures and Tables

**Table 1:** ICP-MS results of TG/Au*_x_*Pt*_y_* catalysts. V_Au_ and V_Pt_ are the adding volumes of HAuCl_4_ and H_2_PtCl_6_ precursor solutions, respectively.

Catalyst	M_Au_ (mg)	M_pt_ (mg)	M_Au_/M_Pt_	Average Size of NPs (nm)	V_Au_ (μL)	V_pt_ (μL)	Au/Pt Atomic Ratio
TG/Au_60_Pt_40_	0.253	0.166	60/40	29.5	90	60	1.53
TG/Au_56_Pt_44_	0.254	0.203	56/44	38.5	90	75	1.26
TG/Au_52_Pt_48_	0.258	0.241	52/48	44.5	90	90	1.07
TG/Au_42_Pt_58_	0.247	0.345	42/58	62.5	90	120	0.72
TG’/Au_53_Pt_47_	0.478	0.412	53/47	45.8	90	90	1.08

**Table 2:** The *J_pf_*, onset potential (*E_onset_*) and ECSA of TG-based catalysts.

Catalyst	*J_pf_* (mA/cm^2^)	*E_onset_* (V vs. RHE)	ECSA (m^2^.g^−1^)
TG	-	-	-
TG/Au_0.258_	-	-	-
TG/Pt_0.241_	1.28	0.74	8.5
TG/Au_60_Pt_40_	3.07	0.63	18.0
TG/Au_56_Pt_44_	4.78	0.60	20.0
TG/Au_52_Pt_48_	5.87	0.59	23.7
TG/Au_42_Pt_58_	7.81	0.64	25.8
TG’/Au_53_Pt_47_	12.92	0.55	40.8

**Table 3:** Comparison of catalyst performance of various graphene-based catalysts. GO: graphene oxide; TG: tubular graphene; NPs: nanoparticles; NA: nanoalloy.

Catalyst	Solutions	*J_pf_* (mA/cm^2^)	Ref.
TG’/Au_53_Pt_47_	0.5 M KOH	12.92	This work
Pt_1_Pd_3_NPs/GO	1 M NaOH	2.73	[68]
PtAuNA/GO	1 M NaOH	7.27	[69]
GO/PtPd	1 M KOH	2.59	[70]
Pt_52_Fe_29_Co_19_@GO-7%	0.5 M H_2_SO_4_	3.42	[14]
PtNi/GO	0.5 M H_2_SO_4_	4.65	[71]

## Data Availability

Data is available upon the reasonable request from the corresponding author.

## Supplementary Materials

The following are available online at https://www.mdpi.com/article/10.3390/nano12101689/s1. Figure S1: (a) The illustration for the preparation of hollow cylindrical structures, (b) SEM image of a typical hollow cylindrical structure of graphene-covered Ni mesh. Figure S2: FE-SEM images of TG/Au*_x_* with different mass loadings of Au NPs. Figure S3: EDS result of the TG/Au_0.258_ catalyst. Figure S4: EDS analyses for the TG/Pt_0.241_ catalyst obtained by scanning on the high coverage (a,b) and low coverage (c,d) of Pt NPs areas. Figure S5: EDS analysis of the TG/Pt_0.241_ before removing Ni. Figure S6: EDS analysis for the TG/Au_52_Pt_48_ catalyst. Right panel shows the elemental distribution of C, O, Au, and Pt. Figure S7: (a–d) TEM-EDS analyses for the TG/Au_52_Pt_48_ catalyst at three different nano-areas. Figure S8: XPS O 1s scans of the TG-based catalysts. Figure S9: SEM images of TG/Au*_x_*Pt*_y_* catalysts obtained with different concentrations of Pt precursor. Figure S10: SEM images at low (upper panels) and high magnification (bottom panels) of the TG’/Au_53_Pt_47_: the distribution of binary AuPt NPs on the outer wall (a) and the inner wall (b) of a graphene tube. Figure S11: CVs of methanol oxidation at cycle number 50 and 200 recorded in 0.5M KOH + 1M CH_3_OH solution of TG/Au_52_Pt_48_ (a) and TG’/Au_53_Pt_47_ (b) catalysts. Table S1: ICP-MS results of TG/Au*_x_* catalysts. V_Au_: the adding volume of HAuCl_4_ precursor solution.
