# Supplementary material for: Free-Standing, Interwoven Tubular Graphene Mesh-Supported Binary AuPt Nanocatalysts: An Innovative and High-Performance Anode Methanol Oxidation Catalyst
Source: Nanomaterials (Basel). 2022 May 16;12(10):1689. doi: 10.3390/nano12101689 (PMC9143563; doi:10.3390/nano12101689)
Supplement: Supplementary file 1 [file nanomaterials-12-01689-s001.zip › nanomaterials-1658678-supplementary.pdf]

nanomaterials

Supplementary Materials

Free-Standing, Interwoven Tubular Graphene Mesh-Supported Binary AuPt Nanocatalysts: An Innovative and High-Performance Anode Methanol Oxidation Catalyst

An T. Nguyen [1,†], Van Viet Tran [1,†], Asnidar Siahaan [1], Hung-Chih Kan [1], Yung-Jung Hsu [2], Chia-Chen Hsu [1,*]

[1] Department of Physics, National Chung Cheng University, Chiayi 621, Taiwan; anhydrit210@gmail.com (A.T.N.); viettran.apc@gmail.com (V.V.T.); asnidarsi-ahaan2014@gmail.com (A.S.); phyhck@ccu.edu.tw (H.-C.K.)
[2] Department of Materials Science and Engineering, National Chiao Tung Yang Ming University,
Hsinchu 30010, Taiwan; yhsu@cc.nctu.edu.tw
* Correspondence: phycch@ccu.edu.tw; Tel.: +886-5-2720411 (ext. 66305)
† These authors contributed equally to this work.

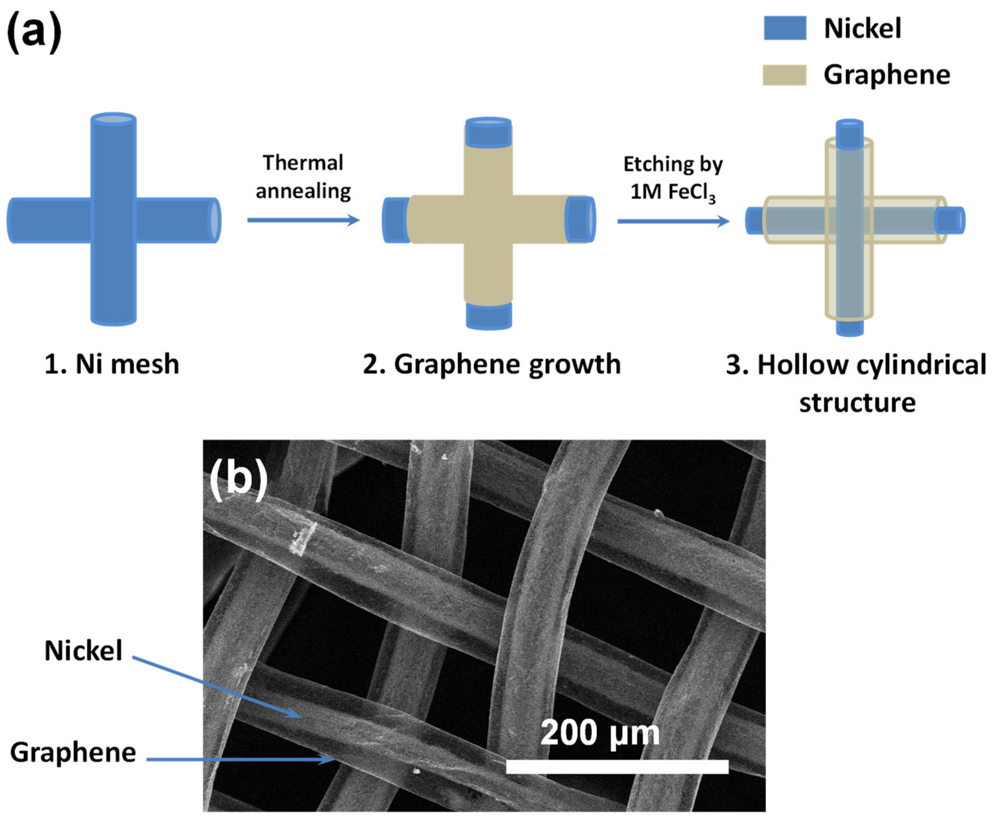

Figure S1. (a) The illustration for the preparation of hollow cylindrical structures, (b) SEM image of a typical hollow cylindrical structure of graphene-covered Ni mesh.

Table S1. ICP-MS results of TG/Au$_x$ catalysts. V_{Au}: the adding volume of $HAuCl_4$ precursor solution.

Catalyst	V_{Au} (µl)	m_{Au} (mg)
TG/Au$_{0.171}$	60	0.171
TG/Au$_{0.258}$	90	0.258
TG/Au$_{0.346}$	120	0.346
TG/Au$_{0.554}$	180	0.554

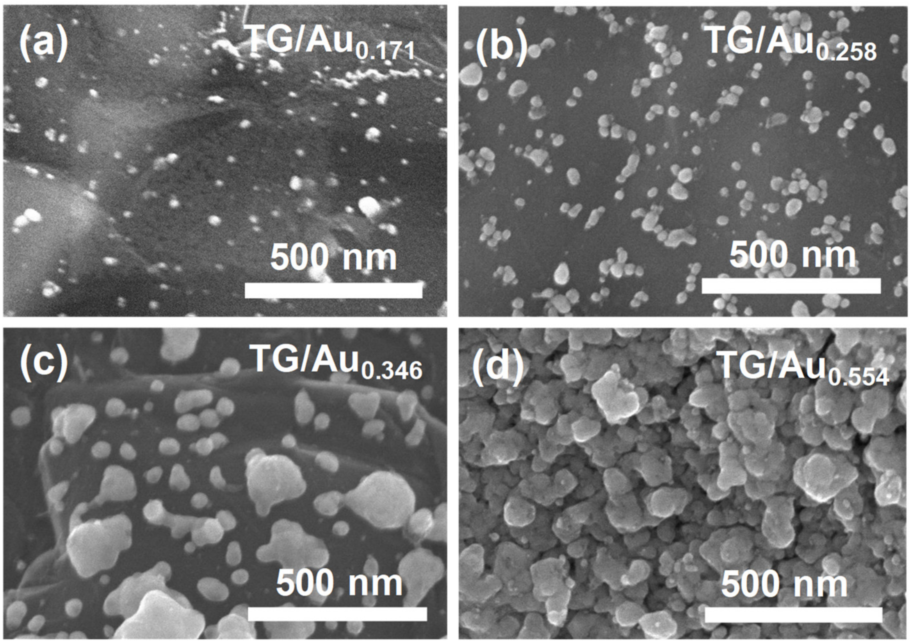

Figure S2. FE-SEM images of TG/Au$_x$ with different mass loadings of Au NPs.

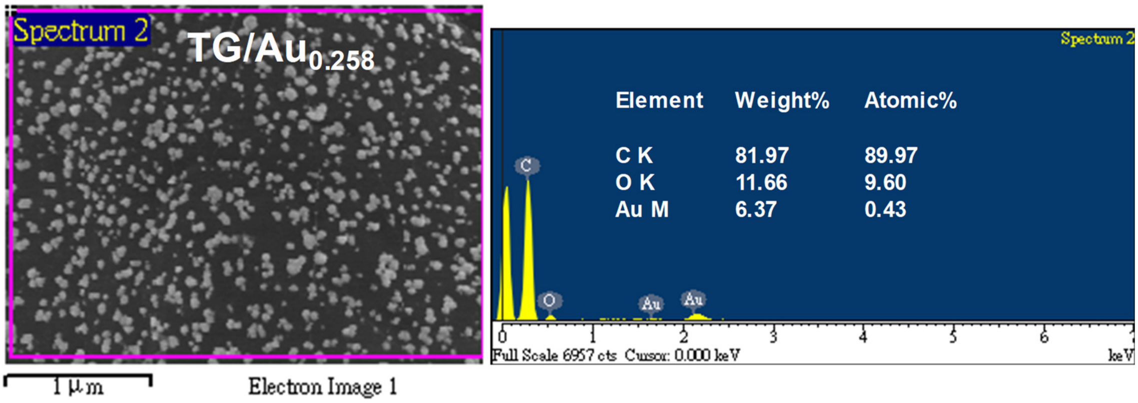

Figure S3. EDS result of the TG/Au$_{0.258}$ catalyst.

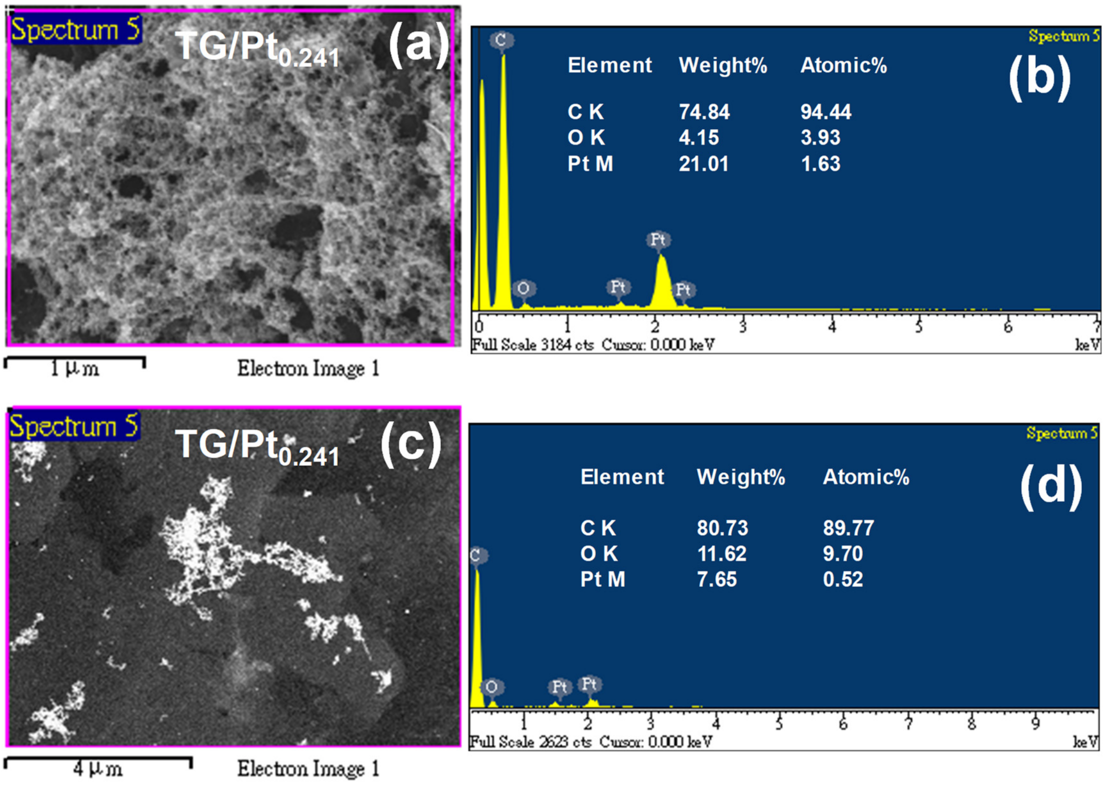

Figure S4. EDS analyses for the TG/$Pt_{0.241}$ catalyst obtained by scanning on the high coverage (a,b) and low coverage (c, d) of Pt NPs areas.

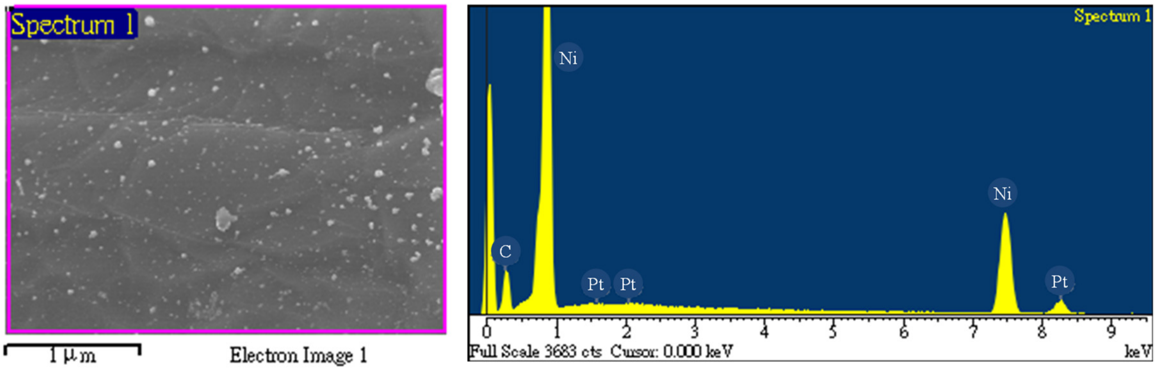

Figure S5. EDS analysis of the TG/$Pt_{0.241}$ before removing Ni. Oxygen was not found in the sample.

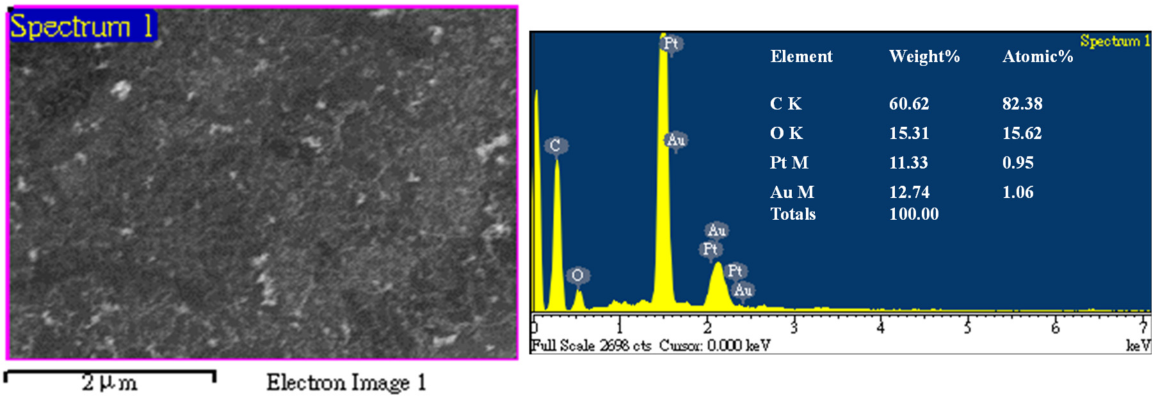

Figure S6. EDS analysis for the TG/$Au_{52}Pt_{48}$ catalyst. Right panel shows the elemental distribution of C, O, Au, and Pt.

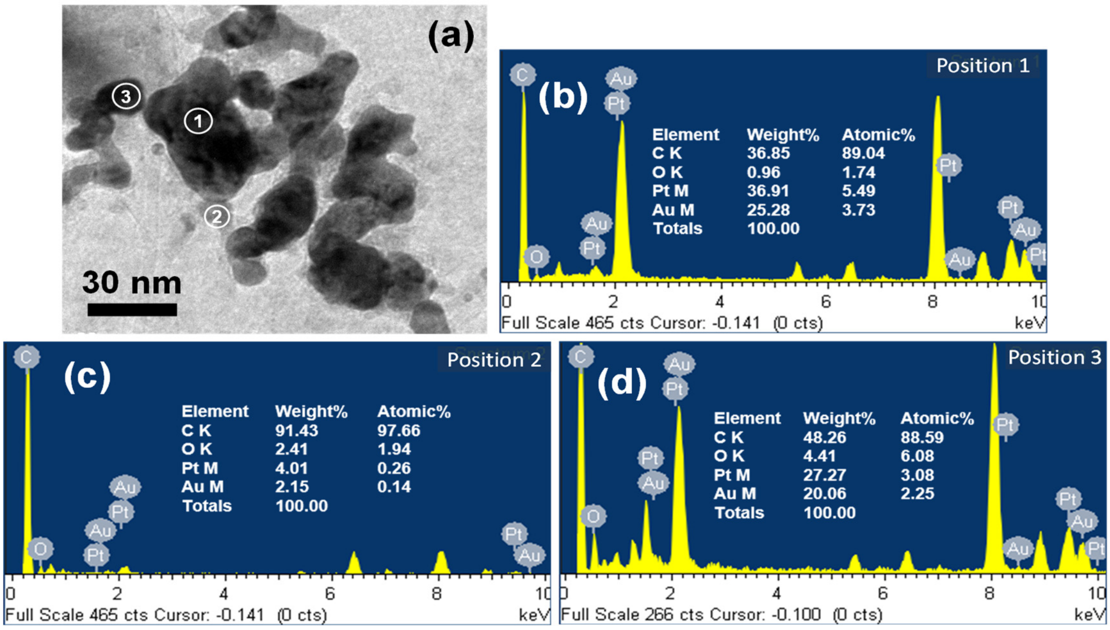

Figure S7. (a-d) TEM-EDS analyses for the TG/$Au_{52}Pt_{48}$ catalyst at three different nano-areas.

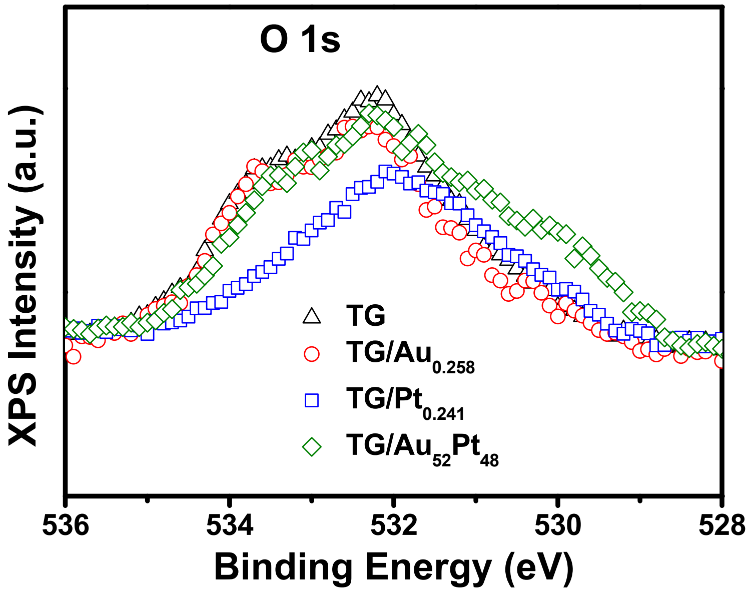

Figure S8. XPS O 1s scans of the TG-based catalysts.

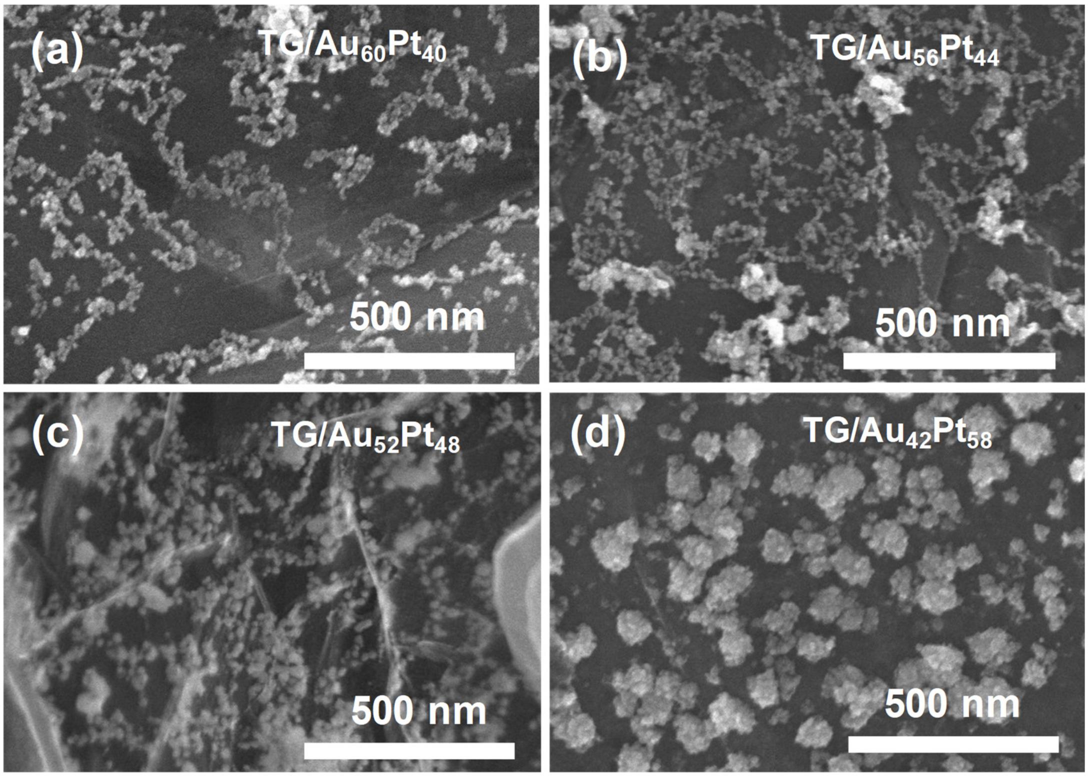

Figure S9. SEM images of TG/Au_xPt_y catalysts obtained with different concentrations of Pt precursor.

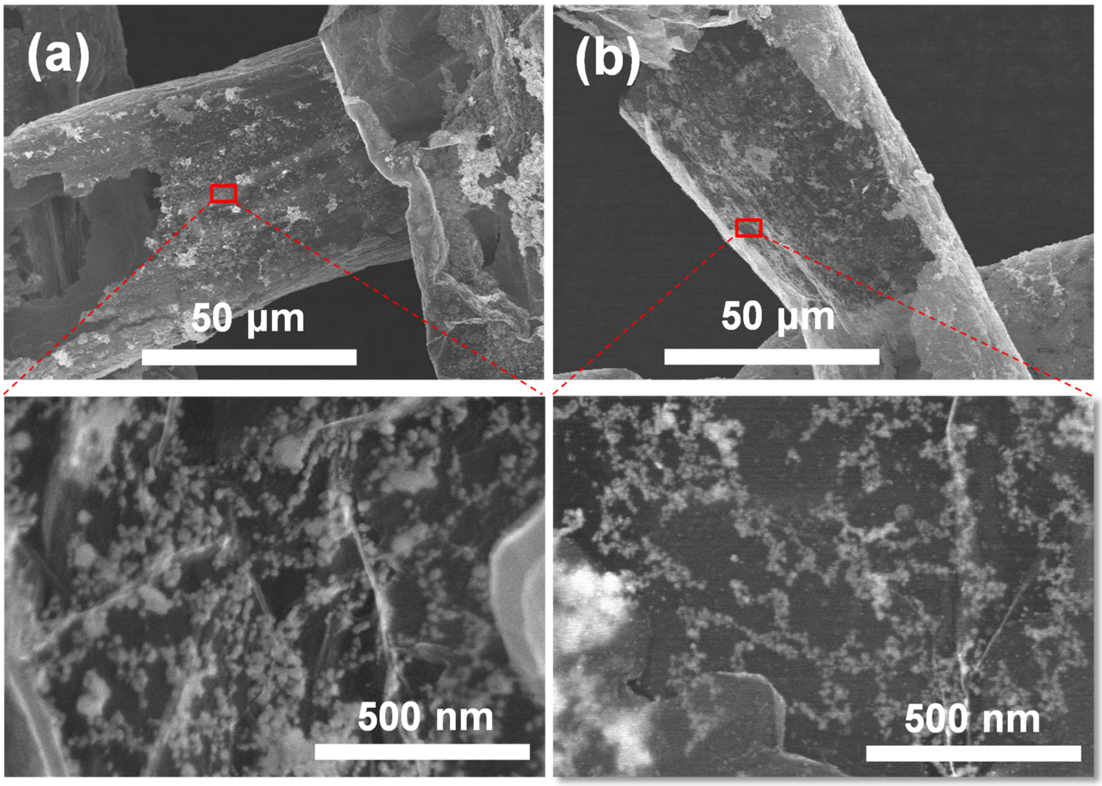

Figure S10. SEM images at low (upper panels) and high magnification (bottom panels) of the TG′/$Au_{53}Pt_{47}$: The distribution of binary AuPt NPs on the outer wall (a) and the inner wall (b) of a graphene tube.

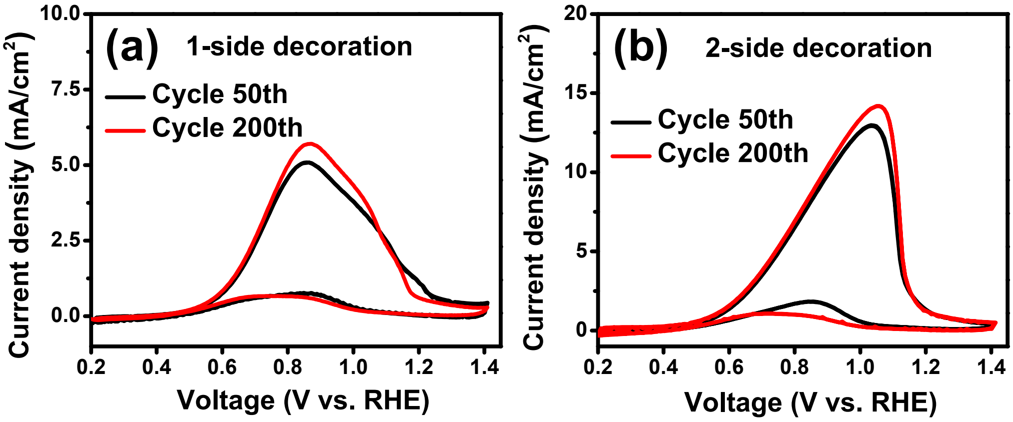

Figure S11. CVs of methanol oxidation at the cycle number of 50 and 200 recorded in 0.5M KOH + 1M CH_3OH solution of the TG/$Au_{52}Pt_{48}$ (a) and TG′/$Au_{53}Pt_{47}$ (b) catalysts.
